# Gastropod-derived haemocyte extracellular traps entrap metastrongyloid larval stages of *Angiostrongylus vasorum*, *Aelurostrongylus abstrusus* and *Troglostrongylus brevior*

**DOI:** 10.1186/s13071-016-1961-z

**Published:** 2017-01-31

**Authors:** Malin K. Lange, Felipe Penagos-Tabares, Tamara Muñoz-Caro, Ulrich Gärtner, Helena Mejer, Roland Schaper, Carlos Hermosilla, Anja Taubert

**Affiliations:** 1 Institute of Parasitology, Justus Liebig University Giessen, Giessen, 35392 Germany; 2 Institute of Anatomy and Cell Biology, Justus Liebig University Giessen, Giessen, 35392 Germany; 3 Department of Veterinary Disease Biology, University of Copenhagen, Frederiksberg C, 1870 Denmark; 4 Bayer Animal Health GmbH, Leverkusen, 51368 Germany

**Keywords:** Gastropod-borne diseases, Metastrongyloidea, Extracellular traps, Lungworm, Innate immune response

## Abstract

**Background:**

Phagocyte-derived extracellular traps (ETs) were recently demonstrated mainly in vertebrate hosts as an important effector mechanism against invading parasites. In the present study we aimed to characterize gastropod-derived invertebrate extracellular phagocyte trap (InEPT) formation in response to larval stages of important canine and feline metastrongyloid lungworms. Gastropod haemocytes were isolated from the slug species *Arion lusitanicus* and *Limax maximus*, and the snail *Achatina fulica*, and exposed to larval stages of *Angiostrongylus vasorum*, *Aelurostrongylus abstrusus* and *Troglostrongylus brevior* and investigated for gastropod-derived InEPT formation.

**Results:**

Phase contrast as well as scanning electron microscopy (SEM) analyses of lungworm larvae-exposed haemocytes revealed ET-like structures to be extruded by haemocytes thereby contacting and ensnaring the parasites. Co-localization studies of haemocyte-derived extracellular DNA with histones and myeloperoxidase in larvae-entrapping structures confirmed classical characteristics of ETs. In vivo exposure of slugs to *A. vasorum* larvae resulted in InEPTs being extruded from haemocytes in the slug mucous extrapallial space emphasizing the pivotal role of this effector mechanism against invasive larvae. Functional larval entrapment assays demonstrated that almost half of the haemocyte-exposed larvae were contacted or even immobilized by released InEPTs. Overall, as reported for mammalian-derived ETs, different types of InEPTs were here observed, i.e. aggregated, spread and diffused InEPTs.

**Conclusions:**

To our knowledge, this study represents the first report on metastrongyloid lungworm-triggered ETosis in gastropods thereby providing evidence of early mollusc host innate immune reactions against invading larvae. These findings will contribute to the better understanding on complex parasite-intermediate host interactions since different gastropod species bear different transmitting capacities for metastrongyloid infections.

**Electronic supplementary material:**

The online version of this article (doi:10.1186/s13071-016-1961-z) contains supplementary material, which is available to authorized users.

## Background

Nowadays increasing attention is being paid to gastropod-borne diseases, both in veterinary and human medicine, on academic, pharmaceutical and clinical practice levels [1–5]. In the last decade canine and feline lungworm species such as *Angiostrongylus vasorum*, *Aelurostrongylus abstrusus* and *Troglostrongylus brevior* are emerging in several countries and spreading into previously non-reported areas [5–12]. Especially the former parasite induces a debilitating disease of the cardiorespiratory system [5, 13] but can also cause neurological, ophthalmic and systemic disease with sometimes life threatening coagulopathies in dogs. The knowledge on how lungworm larval development occurs within gastropod species in vivo is still scarce. Consequently, very little is known on early gastropod-mediated innate immune reactions against these parasites and respective research is urgently needed [12].

In contrast to mammalian species which possess both an adaptive and an innate immune system, gastropods exclusively rely on innate immune responses for pathogen inactivation. Typical mammalian professional mononuclear phagocytes, such as polymorphonuclear neutrophils (PMN), monocytes and macrophages, are lacking in molluscs but are replaced by gastropod-specific phagocytes known as haemocytes (syn. amoebocytes), an invertebrate immune cell subtype that freely circulates within the haemolymph system [14]. Haemocytes were reported to be involved in several physiological functions such as wound repair, coagulation, transport of nutrients and other molecules and intracellular digestion [15, 16]. Overall, this cell type is known as the key player of the molluscan innate immune system [17]. The currently known effector mechanisms of haemocytes are phagocytosis, multicellular encapsulation and cell-mediated cytotoxicity. However, the detailed molecular mechanisms of these immunological processes are not well understood, so far [18–22].

Beginning with the landmark study of Brinkmann et al. [23] which introduced with (N) ETosis a new effector mechanism of PMN, the paradigm of how professional phagocytes fight and kill pathogens has profoundly been changed. ETosis represents a novel type of programmed cell death of PMN and other leucocytes in which the nuclear chromatin and granular proteins are expulsed to the extracellular environment forming thin fibre-like extracellular structures bearing the capacity to capture and inactivate invasive pathogens [24–29]. Besides histones/DNA, several antimicrobial granular molecules, such as defensins, cathelicidins, pentraxin, myeloperoxidase (MPO), calprotectin and lactoferrin [30–32], were reported to be contained within ETs. ETs are cast by different types of leukocytes (e.g. PMN, macrophages, mast cells, monocytes, eosinophils) [33–37], in response to different microorganisms such as bacteria [23], fungi [38], viruses [39] and protozoan/metazoan parasites [29, 40, 41]. ETosis has been reported to occur in numerous vertebrate host types, such as humans, cattle, goats, seals, fish or birds [25, 33, 42–45] as effective innate immune defence mechanism. Consistently, also haemocytes were recently reported to release ETs in invertebrate organisms such as crustaceans and bivalves [46–49]. Extracellular traps have, however, not yet been described in gastropods.

The aim of this study was to investigate for the first time gastropod-derived invertebrate extracellular phagocyte traps (InEPTs) as early host innate immune reactions in different gastropod species (*Limax maximus*, *Arion lusitanicus* and *Achatina fulica*) in response to infective metastrongyloid larvae of *Angiostrongylus vasorum*, *Aelurostrongylus abstrusus* and *Troglostrongylus brevior* in vitro and in vivo. We here present first indications of InEPTs being cast by gastropod haemocytes upon larval exposure. This gastropod immune reaction might have an impact on the development of these obligate heteroxenous lungworm parasites in their intermediate hosts.

## Methods

### Gastropod maintenance

Terrestrial slugs (*A. lusitanicus* and *L. maximus*) and terrestrial giant African snails (*A. fulica*) were bred and maintained in fully-automatized climate incubators (model ECP01E^®^; Snijders Scientific B.V. Tilburg, the Netherlands) under the following controlled conditions: 50% humidity, 10 h of dark/10 h of illumination corresponding to circadian cycles plus 2 h for dusk and dawn each, temperature ranging from 10 to 16 °C (night/day) regarding the slug species and 20–26 °C for *A. fulica*. The slug species were kept in plastic containers supplied with a humidified absorption paper at the bottom, plastic Petri dishes for food and a plastic dim housing area (Tecniplast^®^). Gastropod feedings were performed *ad libitum* twice a week with lettuce leaves (*Lactuca sativa*), cucumber fruits (*Cucumis sativus*), carrot roots (*Daucus carota sativus*), champignons (*Agaricus campestris*), rabbit pellet food (VERSELE-LAGA^®^; CUNIFIT pure) and dry dog food (Purina^®^, Beneful). *Achatina fulica* were maintained in plastic containers on terrarium soil (5 cm height, TerraBasis^®^ and TerraCocoshumus^®^ mixed at 1:1 ratio, JBL) being supplemented with calcium supplement (*ad libitum* 21% calcium, Calcina Calcium Citrat^®^, Canina).

### Generation of axenic metastrongyloid first- (L1) and third-stage (L3) larvae

*Angiostrongylus vasorum* first-stage larvae (L1) were obtained from fresh faeces of experimentally infected red foxes (*Vulpes vulpes*) kindly provided by the Department of Veterinary Disease Biology, University of Copenhagen, Denmark (Danish experimental animal licence no. 2010/561-1914). *Aelurostrongylus abstrusus* and *T. brevior* larvae were recovered from infected cat faeces (kindly donated by S. Rehbein, Merial, Germany). All lungworm larvae were isolated by the classical Baermann funnel technique: 20 g of faeces were placed in a Baermann apparatus equipped with a sieve (aperture 100 μm) and three gauze layers. The funnel was slowly filled with water (20–25 °C) until half of the faecal sample was immersed in water. The apparatus was incubated at room temperature (RT) for 24 h during which the larvae migrated from the faeces into the water owing to positive hydrotaxis and sedimented. By carefully opening the clamp 5 ml sediment were collected in 15 ml conical tubes (Greiner). Then the larvae were concentrated and freed from faecal contamination by Percoll gradients (Merck) as reported elsewhere by Graeff-Teixeira et al. [50].

*Angiostrongylus vasorum* third-stage larvae (L3) were generated from experimentally infected *L. maximus* slugs at 30 days post-infection (p.i.) *via* artificial digestion. The experimental procedure was the following: slugs were cut in small pieces and digested in digestion solution [1 l contained 10 g pepsinogen powder 2000 FIP-U/g (Robert Kind), 8.5 g NaCl (Carl Roth), 30 ml HCl 37% (Carl Roth), distilled water ad 1 l]. The digestion was performed in 50 ml Falcon tubes (Greiner) under constant shaking (4 h, 40 °C). The digested samples were sieved firstly through a 300 μm-metal sieve (Retsch) to remove undigested material and then through a 25 μm-metal sieve (Retsch). The remnants of the last sieving were transferred to 15 ml Falcon tubes and centrifuged (400× *g*, 10 min). The pellets were resuspended and examined microscopically (Leica light microscope at 4× and 20× magnification). Viable metastrongyloid larvae were carefully collected by pipetting (Pasteur pipette, Hirschmann GmbH & Co. KG).

To remove any bacterial contaminants and to achieve axenic L1 and L3, larvae were incubated for 10 min in 10 ml sodium hypochlorite solution (0.5% *v*/*v*; Carl Roth) prepared with sterile phosphate-buffered saline (PBS) as previously described elsewhere [51]. Additionally, the larvae were washed twice (250× *g*, 5 min, 20 °C) in sterile PBS supplemented with 3% penicillin (500 U/ml; Sigma-Aldrich) and streptomycin (500 μg/ml; Sigma-Aldrich, Darmstadt, Germany). Axenic larvae were prepared two days before InEPT-related experiments in order to conserve a high larval viability.

### Slug exposure to *Angiostrongylus vasorum* first-stage larvae (L1) in vivo

In order to evaluate the earliest time point of gastropod-mediated innate immune reactions directed against invading metastrongyloid nematodes, a living juvenile slug (*L. maximus*) of *c*.5 mm of length was exposed to viable axenic L1 of *A. vasorum.* Therefore, the juvenile slug was allocated on a cover slip of 10 mm diameter in a 12-well-plate and confronted to 500 L1 axenic *A. vasorum* larvae diluted in 1 ml sterile PBS. After 10 min of incubation the juvenile slug was cryo-anesthetizied (-20 °C, 5 min), fixed in 2.5% glutaraldehyde (60 min, RT, Merck) and processed for scanning electron microscopy (SEM).

### Scanning electron microscopy (SEM)

The fixed samples were post-fixed in 1% osmium tetroxide (Merck, Darmstadt, Germany), washed in distilled water, dehydrated, critical point dried by CO_2_-treatment and sputtered with gold. Thereafter, the samples were examined with a Philips XL30 scanning electron microscope at the Institute of Anatomy and Cell biology, Justus Liebig University Giessen, Germany.

### Haemolymph extraction and in vitro culture of gastropod haemocytes

Gastropods were subjected to a 48 h fasting period and cryo-anesthetized (40 min on ice) before haemocyte isolation was performed. A modified serum-free haemocyte collection solution [77% RPMI, 20% anticoagulant buffer (98 mM NaOH, 186 mM NaCl, 1.7 mM EDTA and 41 mM citric acid, pH 4.5) and 3% penicillin/streptomycin (Sigma-Aldrich, penicillin 10,000 U/ml, streptomycin 10 mg/ml)] according to Stoepler et al. [52] was injected into each cryo-anesthetized gastropod corresponding to 10% of its body weight. Thereafter, slugs were cryo-anesthetized (20 min on ice) again before euthanasia was performed via fast decapitation as described elsewhere [53]. The haemolymph samples were collected by aspiration from decapitated slugs and mixed immediately with 200 μl sterile culture medium composed of penicillin- (500 U/ml; Sigma-Aldrich) and streptomycin- (500 μg/ml; Sigma-Aldrich) supplemented RPMI 1640 medium (Gibco). The haemolymph of *A. fulica* was collected *via* insertion of a needle with syringe close to the pneumostome as described elsewhere [54] and treated the same way as the haemolymph of slugs. The cells were washed thrice (250× *g*, 5 min) and counted in a Neubauer haemocyte chamber. The gastropod haemocytes were co-cultured with axenic L1 of *A. vasorum*, *A. abstrusus* and *T. brevior* as well as with L3 of *A. vasorum* (Table 1) on poly-_L_-lysine (Sigma-Aldrich) pre-coated coverslips at a ratio of 200:1 (RT, in the dark 30 min). Thereafter, the samples were fixed either in 2.5% glutaraldehyde (RT, Merck) for SEM analysis or in 4% (*w/v*) paraformaldehyde for fluorescence microscopic analyses and stored at 4 °C until further used. For negative controls cells were treated the same way as mentioned above. Instead of lungworm larvae only culture medium was added (*n* = 5).

**Table 1:** Overview of conducted nematode/gastropod confrontations

	*Limax maximus*	*Arion lusitanicus*	*Achatina fulica*
*Angiostrongylus vasorum*	L1		L1 and L3
*Aelurostrongylus abstrusus*	L1	L1	L1
*Troglostrongylus brevior*	L1		
In vitro	×	×	×
In vivo	×		
SEM	×		×
Phase contrast microscopy	×	×	×
Immunofluorescence microscopy		×	×

### Characteristics to determine ET morphology

According to Muñoz-Caro et al. [29], Schauer et al. [55] and Hakkim et al. [56] ET structures were described referring to their appearance as “diffuse” ETs (*diff*ETs), “spread” ETs (*spr*ETs), “aggregated” ETs (*agg*ETs) and ETs that display an anchor-like effect. “Diffuse” ETs (*diff*ETs) are described as consisting of a globular and compact form with a size of 25–28 nm diameter, whereas “spread” ETs (*spr*ETs) were observed consisting of smooth and elongated web- like structures composed exclusively by thin fibers with a diameter of 15–17 nm. Furthermore, so-called “aggregated” ETs (*agg*ETs), according to Schauer et al. [55] were displayed as large clusters with a “ball of yarn”-like clumpy and massive appearance involving a high number of immune cells. These ET structures appeared with sizes larger than 50 μ m in diameter. A reduction in larval forward-motility due to fine ET structures (*spr*ETs) which are connected to the *agg*ETs entrapping larvae and clearly hampering larval motility was referred to as an anchor-like effect.

### Immunofluorescence analyses of InEPTs

For DNA staining the samples were stained by DAPI according to Martinelli et al. [57] and Lippolis et al. [58]. For the detection of nuclear histones and MPO within haemocyte-derived ET structures the following specific monoclonal antibodies were used: anti-histone (H1, H2A/H2B, H3, H4; Merck Milipore, MAB3422, 1:1000) and anti-MPO (Biorbyt; orb11073, 1:1000). The samples were washed twice in PBS and blocked with bovine serum albumin (BSA, 2%, Sigma-Aldrich) and reacted with primary antibody solutions (1 h, RT). After two washings, the samples were incubated for 1 h (RT, in the dark) either in Alexa Fluor-conjugated goat anti-mouse monoclonal antibody solution (mouse clone, 1:1000, Thermo Fisher Scientific, for histone detection) or in goat anti-rabbit antibody solution (1:1000, Thermo Fisher Scientific, for MPO detection). The samples were then washed in PBS, mounted in anti-fading buffer (Mowiol^®^; Sigma-Aldrich) and examined microscopically (Olympus IX81^®^ phase contrast microscope equipped with a digital camera and the analySIS^®^ software).

### InEPT-entrapment assay

Gastropod haemocytes (*n* = 3, 1 × 10^4^) were seeded on poly-_L_-lysine pre-coated coverslips and exposed to axenic *A. vasorum*-L1 (50 larvae/sample) in 300 μl RPMI medium 1640 (1% penicillin/streptomycin, without phenol red, Sigma-Aldrich, for 30 min at RT). Thereafter, the coverslips were fixed (4% paraformaldehyde) and InEPT-entrapped larvae were counted by using an inverted DMIRB^®^ phase contrast microscope (Leica). Larvae were considered as entrapped when cell aggregates of at least two haemocytes or stretches of different kinds of ETs (*agg*InEPTs, *spr*InEPTs or *diff*InEPTs) were in direct contact with the larvae. The data were expressed as percentage of entrapped L1 relative to the total amount of *A. vasorum* L1.

Haemocytes of three gastropod species (*Limax maximus*, *Arion lusitanicus* and *Achatina fulica*) were confronted to three nematode species (*Angiostrongylus vasorum*, *Aelurostrongylus abstrusus* and *Troglostrongylus brevior*) of different parasitic stages (L1 and L3) in in vitro and in vivo experiments with the use of different visualisation methods (SEM, phase contrast microscopy, immunofluorescence microscopy).

## Results

### Metastrongyloid lungworm larvae induce gastropod InEPTs in a parasite species- and stage-independent manner

Microscopic analyses revealed that exposure of first- and third-stage larvae of *A. vasorum* to gastropod haemocytes trigger the formation of ET-like structures indicating a stage-independent process (Additional file 1) since both larval stages (L1, L3) were attacked by InEPTs. To account for parasite-specificity we also tested *A. abstrusus-* and *T. brevior-*L1 for their capability to induce InEPTs and indeed both parasite species triggered InEPT formation in exposed haemocytes (see Additional file 1; Additional file 2: Figure S1; Additional file 3: Figure S2). These data clearly provide evidence against a parasite-specificity of metastrongyloid-triggered InEPT formation highlighting the capacity of gastropod haemocytes to equally react to different lungworm parasites therefore the reaction is species-independent.


Additional file 1: Metastrongyloid larvae entrapment by gastropod-derived InEPTs. (MOV 5119 kb)


**Fig. 1:**
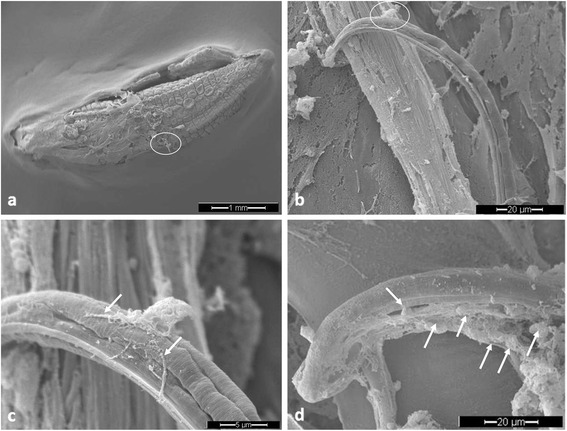
In vivo InEPT formation in *Limax maximus* as early response against living *Angiostrongylus vasorum* larvae. **a** Scanning electron microscopy overview on the total slug, *circle* shows the area of interest which is magnified in (**b**). **b** *Angiostrongylus vasorum* larvae on the slug surface, *circle* shows area of interest which is magnified in (**c**). **c** Haemocytes in tight contact to lungworm larvae releasing delicate ET-like fibres (*spr*InEPTs, *arrows*). **d** Haemocytes (*arrows*) on the surface of the slug attached to a L1 larvae releasing delicate web-like structures (*diff*InEPTs) which cover the surface of the larvae

**Fig. 2:**
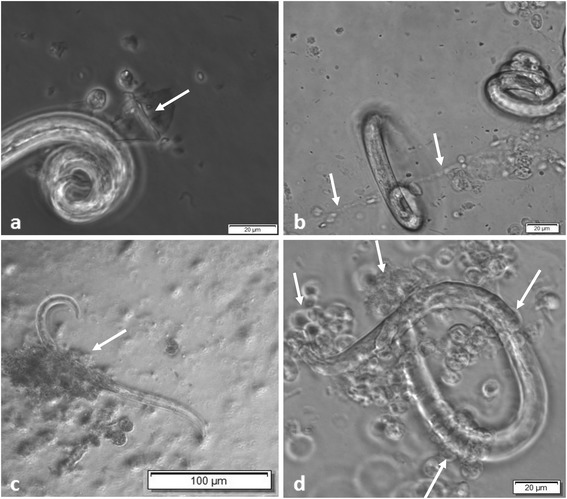
Early ET formation against metastrongyloid lungworm larvae. **a** *Achatina fulica* haemocytes acting against *A. vasorum* L1, spread ETs (*spr*InEPTs, *arrow*, 30 min). **b** *Achatina fulica* haemocytes acting against *A. vasorum* L1, spread ETs (*spr*InEPTs, arrows, 30 min). **c** *Arion lusitanicus* haemocytes acting against *A. abstrusus* L1, aggregated ETs (*agg*InEPTs, *arrows*, 60 min). **d** *Achatina fulica* haemocytes against *A. vasorum* L1, aggregated ETs (*agg*InEPTs, *arrows*, 60 min)

**Fig. 3:**
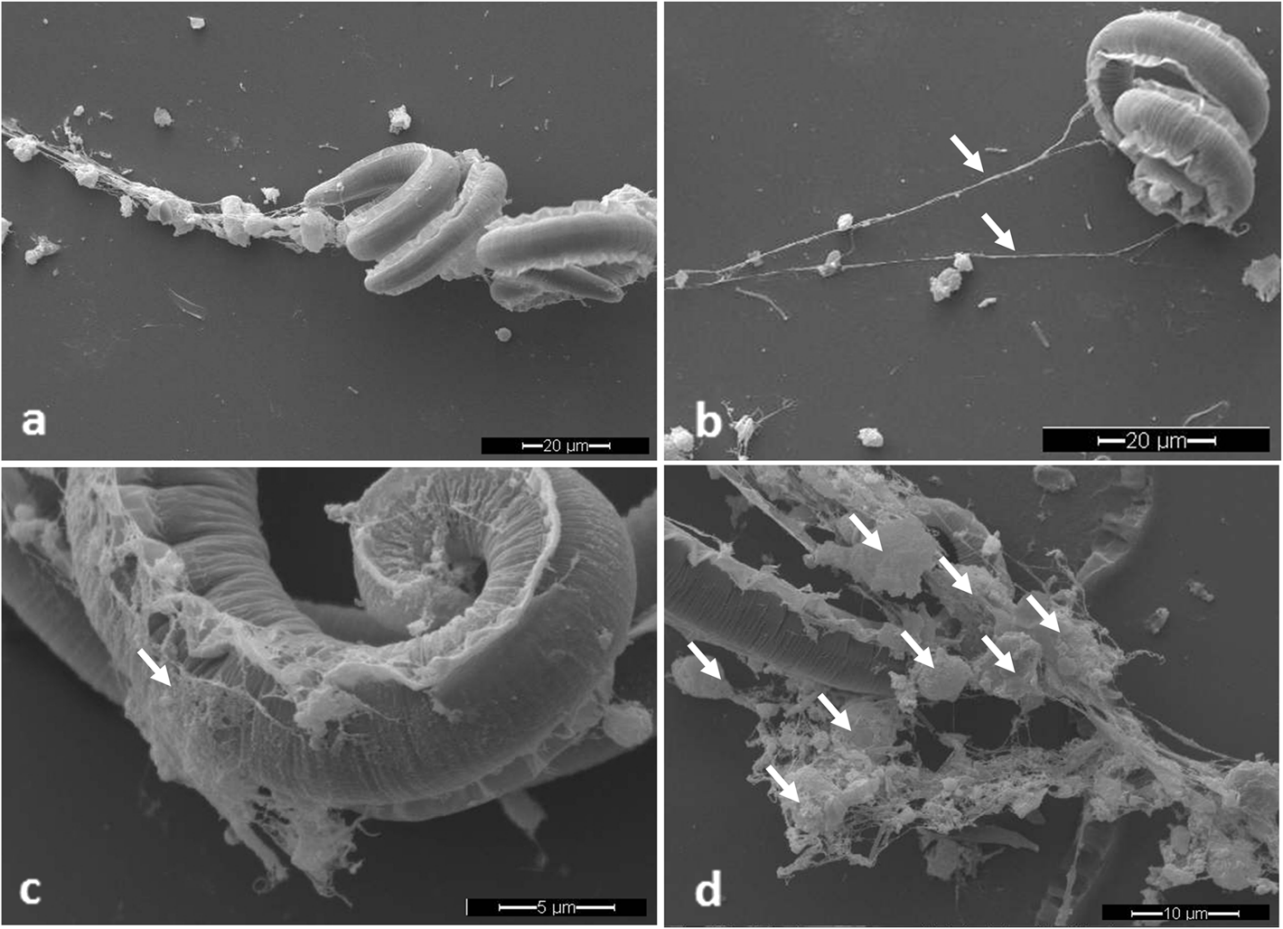
ETs formed by gastropod haemocytes after the confrontation with *Angiostrongylus vasorum* L1. Scanning electron microscopy analysis revealed InEPTs being formed by haemocytes of *A. fulica* co-cultured with L1 showing different types of InEPTs **a** two larvae entrapped by a chain of InEPTs and haemocytes, **b** spread InEPTs (*spr*InEPTs, *arrows*), **c** diffused InEPTs (*diff*InEPTs, *arrows*), **d** haemocytes (*arrows*) forming aggregated InEPTs (*agg*InEPTs)

Overall, we detected typical ‘diffuse’ InEPTs (*diff*InEPTs) which are reported to be composed of a complex of extracellular decondensed chromatin adorned with antimicrobial histones and proteins and show a rather compact form with sizes of 20–30 μm in diameter [29]. Additionally, ‘spread’ InEPTs (*spr*InEPTs) were here observed consisting of smooth and extremely elongated web-like extracellular structures (Figs. 1, 2 and 3). Interestingly, in the case of *A. vasorum* larvae InEPTs were consistently observed in close proximity to the alae of the larvae indicating this structure as a possible target of haemocytes (Fig. 3d). Furthermore, the presence of metastrongyloid-triggered ‘aggregated’ InEPTs (*agg*InEPTs), in accordance with Schauer et al. [55] and Muñoz-Caro et al. [29], were also detected as large clusters of ET-like structures with a clumpy morphology reaching sizes larger than 50 μm in diameter and involving a high number of haemocytes (Fig. 3c). Especially the combination of these structures with *spr*InEPTs appeared strong enough to hamper larvae from movements by entangling them (see Fig. 3 and Additional file 1). Thus, anchor-like effects originating from few haemocytes captured lungworm larvae mainly at one end of the body and were often connected to *agg*InEPTs. Although larvae moved rigorously to escape they seemed entrapped in these InEPT structures (see Fig. 3 and Additional file 1).

**Fig. 4:**
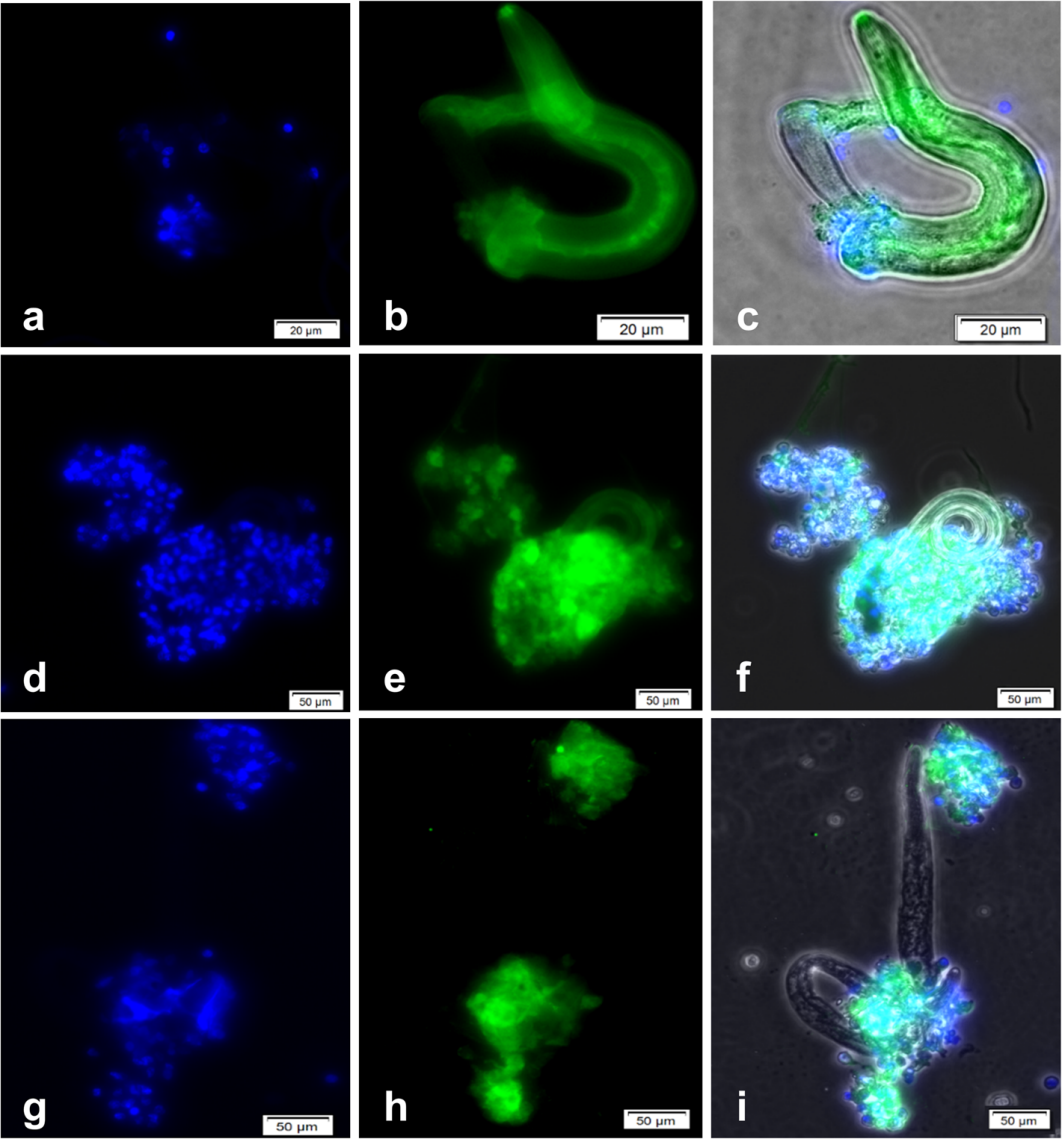
Co-localisation of DNA, histones (H1, H2A/B, H3, H4) and MPO in InEPT structures. Co-cultures of *A. lusitanicus* haemocytes with *A. abstrusus* (**a**-**c**) and *A. fulica* haemocytes *with A. vasorum* L1 (**d**-**i**) induced the formation of ETs with the classical constituents: DNA (*blue* DAPI, **a**, **d**, **g**), histones (*green*, **b**) and MPO (*green*, **e**, **h**). The merges illustrate the larvae and the constituents of the ETs derived from haemocytes (**c**, **f**, **i**)

Irrespective of the InEPT types, antibody-based experiments revealed that gastropod-derived InEPTs were composed of extracellular chromatin being decorated with histones (H1, H2A/H2B, H3, H4) and MPO-like proteins thereby confirming classical characteristics of ETs (Fig. 4).

### Metastrongyloid larvae-driven InEPTosis is (intermediate) host-independent

Lungworm parasites are known to infect a broad panel of gastropod intermediate host species. In order to evaluate whether metastrongyloid-mediated InEPTosis is an intermediate host-specific event or rather represents a general effector mechanism accounting for most slugs and snail species, we also analysed this parasite-triggered InEPT formation in three different gastropod species, i.e. in *A. lusitanicus*, *L. maximus* and *A. fulica* (Table 1). Overall, haemocytes of all three gastropod species cast InEPTs in response to vital metastrongyloid lungworm larvae which argues against a strict intermediate host-specific reaction.

### Gastropod InEPTs entrap *Angiostrongylus vasorum* larvae

**Fig. 5:**
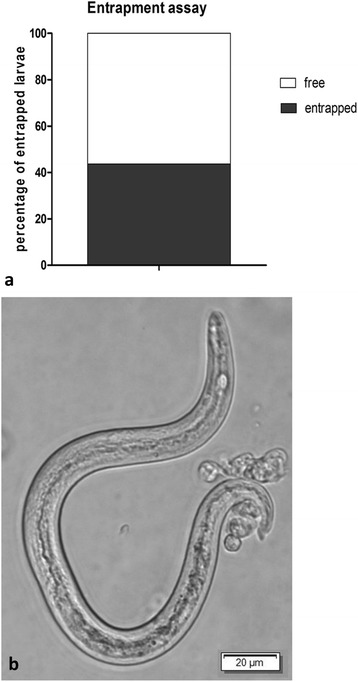
Entrapment assay of *Angiostrongylus vasorum* L1 exposed to haemocytes of *Achatina fulica*. Co-cultivation of haemocytes of *A. fulica* were seeded on poly-_L_-lysine pre-coated coverslips and exposed to *A. vasorum* first-stage larvae. Larvae were considered as entrapped when cell aggregates of at least two cells were in contact with the larvae (**b**). The data are expressed as percentage of attacked L1 relative to the total amount of L1 (**a**)

In order to analyse the efficacy of haemocyte-derived InEPTs to entrap viable *A. vasorum* L1, we established a quantitative parasite-entrapment assay by exposing haemocytes to *A. vasorum*-L1 and thereafter counting larvae that were found either entrapped within extruded InEPT structures or attached by haemocyte agglomerates. Functional parasite-entrapment experiments revealed a proportion of 41% of *A. vasorum* larvae to be in contact and most probably immobilized by InEPT structures (see Fig. 5). These results indicate a rather high efficacy of gastropod InEPT-mediated entrapment considering that almost every second larva was ensnared and most probably hampered from intermediate host invasion.

### In vivo slug exposure to *Angiostrongylus vasorum*-L1 results in extrapallial InEPT formation

Given that slug haemocytes are known to be also present in the peripheral extrapallial fluid [17, 59, 60], we here intended to visualize the first contact of invading parasites on the surface of the slug. In vivo SEM evaluation of this earliest host innate immune reaction against *A. vasorum*, clearly demonstrated that extrapallial haemocytes were present in the slug mucus and firmly attached to the cuticle of invading larvae (Fig. 1). As a defence mechanism, some of these haemocytes cast ET-like structures towards the nematode cuticle (Fig. 1). These ET-like extracellular structures were also detected in a more spread, net-like pattern originating from more than one haemocyte on the surface of the larvae. In line with previous descriptions of mammalian ET morphologies [29] we here found both, smooth and elongated extracellular structures composed exclusively of thin fibres (Fig. 1c) and rather web-like spread forms (Fig. 1d). Given that the experiment was performed already 10 min after the first contact between parasite and intermediate host these data clearly confirm that InEPT induction represents a very fast and early effector mechanism that even precedes pathogen invasion into the slug corpus, i.e. in the extrapallial space (mucus) of gastropods.

In the negative controls no cell aggregates were observed in four of the five experiments. Only in one coverslip cells formed occasionally aggregates without typical ET-like fibres.

## Discussion

To our knowledge, this study delivers first evidence on the release of ETs as part of the early innate immune response of gastropod haemocytes acting against metastrongyloid lungworm larvae. So far, ETosis was reported only in few other invertebrate hosts, such as oysters (*Crassostrea gigas* [46, 61]), the shore crab *Carcinus meanas* and the blue mussel *Mytilus edulis* [47] and shrimp [62]. ETs mainly consist of chromatin, nuclear histones (H1, H2A/H2B, H3, H4) and granular components, such as NE, MPO, lactoferrin, pentraxin and gelatinase amongst others [24, 26, 40]. In accordance to recent findings on InEPT reactions of the shore crab [47] we here confirmed the typical decoration of externalised chromatin of InEPTs by co-localization with histones (H1, H2A/H2B, H3, H4) and MPO on gastropod-derived InEPTs being cast against lungworm larvae. These results are also in accordance to reports on parasite-triggered ETosis in the mammalian system, which demonstrated histones and MPO as main components of ETs in vitro as well as in vivo [25, 29, 33, 40, 43, 63–65]. Regarding the effect of deoxyribonuclease (DNase) treatment in mammalian ET-formation Behrendt et al. [25] described the disintegration of NETs, highlighting the backbone-nature of chromatin. Moreover, DNase treatment on parasite-triggered ET formation illustrated that entrapped *Besnoitia besnoiti* tachyzoites were neither killed by ETs since their host cell infectivity was entirely restored upon DNase treatment [37, 64]. Until now not much is known on ET-evasion-mechanisms of parasites [40] but Guimarães-Costa et al. [66] mentioned the ability of *Leishmania* parasites to escape NET-mediated killing by producing nucleases. For future investigation it would be furthermore of interest to conduct in vitro assays with known ETosis inhibitors to proof that InEPT formation in gastropods is controlled and deliberate and is not a consequence of parasite-haemocyte damage that allows the nuclear material to escape passively from the host cell.

During the life-cycle of most metastrongyloid nematodes of domestic animals and humans, exogenous metastrongyloid L1 must actively invade terrestrial mollusc intermediate hosts to fulfil further development into infective L3 [2, 67]. By performing this obligate step of the life-cycle, larvae will become potential targets of the mollusc innate immune system. Mollusc haemocytes, which correspond to mammalian professional phagocytes, such as neutrophils, monocytes, macrophages, are known to be actively recruited to the site of infection and additionally to actively migrate into the gastropod mucous extrapallial space [17, 59, 60]. The current in vivo and in vitro data demonstrate that gastropod-derived ETosis is a conserved, ancient and efficient effector mechanism as already demonstrated elsewhere [24, 25, 33, 40, 64, 68]. So far, several reports exist on protozoan-triggered ETosis [25, 42, 43, 63–65, 68–70] whilst only few data are available on metazoan-triggered ETosis. First ever reported metazoan-induced ETosis was reported on trematodes. As such, *Schistosoma japonicum* has recently been identified as potent ET-inducer in vitro and in vivo [41]. Besides the here described lungworms, ET-inducing nematodes species include only *Strongyloides stercoralis* [71] and *Haemonchus contortus* [29].

The current data suggest that gastropod-derived InEPT formation is a rather general effector mechanism against metastrongyloid parasites. Thus, gastropod-derived InEPT release occurred irrespective of the parasite species (i.e. *A. vasorum, A. abstrusus*, *T. brevior*), the parasite stage (L1 *vs* L3) and of the haemocyte origin (i.e. *A. lusitanicus*, *L. maximus*, *A. fulica*). In agreement, data on different protozoans, such as *Leishmania* spp. [69] and *Eimeria* spp. [65], also indicated ETosis as a parasite species- and stage-independent defence effector mechanism. In addition, *Eimeria bovis-* and *Cryptosporidium parvum-*triggered ETosis has recently also been reported as host origin-independent innate immune reaction [65]. It is worthwhile to mention, that the efficacy of ET-mediated nematode entrapment differed between different parasite and host species. Thus, in the current work, up to 41% of larval entrapment was observed which is considerably lower compared to *H. contortus* NET-mediated larval entrapment in the mammalian system [29]. This survey represents the first report on InEPTs extruded by haemocytes against lungworms and these data will serve as a base line for further in depth investigation.

As an interesting feature, we observed the in vitro-release of different types of InEPTs, such as *diff*InEPTs, *spr*InEPTs and *agg*InEPTs and anchor-like effects upon contact with larvae of the lungworm species *A. vasorum*, *A. abstrusus* and *T. brevior*. Anchor-like nematode entrapment performed by gastropod haemocytes is consistent to previously reported NETs acting against *Haemonchus contortus* [29]. At least two types of InEPTs (i.e. *diff*InEPTs, *spr*InEPTs) were additionally found to occur in vivo within the mucus of slugs acting against actively invading *A. vasorum* L1*.* All types of InEPTs promoted efficient ensnarement of the larvae since almost half of the larvae were found immobilized by these extracellular structures*.* However, in contrast to other invertebrate ET reactions [48], lethal effects of InEPTs could not be observed even within a prolonged incubation period (4 h). Consequently, the tight immobilization of larvae seems to represent the key mechanism of larval-induced InEPTs. Thus, we postulate that even though InEPT formation themselves do not seem capable of killing nematode larvae they might entrap them and prevent active invasion and further migration through the mollusc body. Moreover, it seems plausible to assume that InEPT-entrapped larvae might become exposed to other recruited haemocytes [24, 40].

Importantly, *A. vasorum*-mediated InEPT induction already occurred within the mucous surface of the gastropod tegument, i.e. even before larval host penetration, thereby representing the earliest possible time point of parasite-intermediate host interactions. Given that generally only few larvae are detected in gastropod intermediate hosts [72], this early attack may thus have a high impact on the dispersal of the disease. So far, it is still unclear how slug haemocytes recognize the larvae in terms of ETosis and which parasite-derived molecules are involved in this process. Nonetheless, the current data on vital metastrongyloid larvae suggest that molecules are most probably present on the cuticle, but it cannot be excluded that also excretory/secretory molecules might be able to act as triggers of InEPT formation. In this context, haemocyte-derived sensing of the larvae might also be a matter of size since Branzk et al. [27] reported on the ability of professional phagocytes to distinguish between small- and large-sized pathogens and to selectively cast ETs in response to large pathogens. Recently, physical properties of particles, such as shape and rigidity, have also been demonstrated to influence on the type of response of phagocytes [73]. These mechanisms may apply for ET induction by large metazoan parasites such as lungworms, *S. japonicum*, *S. stercoralis* and *H. contortus*, especially since phagocytosis is ineffective against these large multicellular parasites. In agreement with Muñoz-Caro et al. [29], the main two functions of gastropod-derived InEPTs acting against large nematode larval stages may be firstly to reduce proper larval invasion and dispersal within the gastropod host and secondly to expose InEPT-entrapped larvae to other immune cells in vivo. A partial or even complete blockage of larval invasion by gastropods could result in diminished parasite burden and thereby significantly influence the intermediate host capacity of certain species. Considering the fact that ETs are mainly composed by antimicrobial histones, peptides and proteins, we also speculate that InEPTs might serve to administer haemocyte-derived anthelmintic compounds to the larval cuticle or in the near vicinity of entrapped parasites. As such, in the mammalian system, eosinophil-derived neurotoxin (EDN) will be more efficient if localized close to the larvae [29]. The release of EDN might restrict the larval motility thereby preventing further development and allowing adhesion of eosinophils to discharge toxic granule contents on the larval surface [74]. As such, eosinophil-derived ETosis was recently demonstrated as a response to the nematode *H. contortus* [29]. However, whether gastropod haemocyte granules also contain EDN-like molecules remains to be ascertained in the future. Nonetheless, our data strongly suggest mollusc InEPTs as an important effector mechanism of haemocytes acting against metastrongyloid lungworms of dogs and cats.

## Conclusions

In summary, we postulate that InEPTosis might diminish the establishment and development of metastrongyloid larvae within the obligate gastropod intermediate host by immobilizing the larvae and hampering them from migration through the tegument or body. Mollusc InEPTs may further facilitate the exposure and attack of entrapped large-sized parasites by other immunocompetent haemocytes. In consequence, lungworm larvae-triggered InEPTosis will also have an impact on the in vivo situation and may therefore influence the intermediate host capacities of different slug/snail species. Comparative studies on zoonotic relevant parasites, such as *A. cantonensis* and *A. costaricensis*, are urgently required to gain more knowledge on gastropod-borne parasitoses. Thus, we call for more mollusc-based investigations in order to better understand the actual spread of gastropod-borne diseases in humans, domestic as well as wild animals.

## Additional files

Additional file 2: Figure S1. Metastrongyloid larvae of *Troglostrongylus brevior* and *Aelurostrongylus abstrusus* attacked by mollusc haemocytes. Early ET formation against L1 of *Troglostrongylus brevior* confronted to haemocytes of *Limax maximus* analysed with contrast phase microscopy. (DOCX 376 kb)
Additional file 3: Figure S2. Early ET formation against L1 of *Aelurostrongylus abstrusus* confronted to haemocytes of *Limax maximus* analysed with contrast phase microscopy. (DOCX 1533 kb)

## Abbreviations

*agg*InEPTs: Aggregated InEPTs
DAPI: 4′,6-diamidino-2-phenylindole
*diff*InEPTs: Diffused InEPTs
DNA: Deoxyribonucleic acid
DNase: Deoxyribonuclease
EDN: Eosinophil-derived neurotoxin
EDTA: Ethylenediaminetetraacetic acid
ET: Extracellular traps
InEPT: Invertebrate extracellular phagocyte traps
L1: First-stage larvae
L3: Third-stage larvae
MPO: Myeloperoxidase
NE: Neutrophil elastase
PBS: Phosphate-buffered saline
PMN: Polymorphonuclear neutrophils
RPMI: Roswell Park Memorial Institute medium
SEM: Scanning electron microscopy
*spr*InEPTs: Spread InEPTs

## References

[1] Traversa D, Guglielmini C (2008). Feline aelurostrongylosis and canine angiostrongylosis: a challenging diagnosis for two emerging verminous pneumonia infections. Vet Parasitol.

[2] Spratt DM (2015). Species of *Angiostrongylus* (Nematoda: Metastrongyloidea) in wildlife: A review. Int J Parasitol Parasites Wildl.

[3] Aghazadeh M, Jones MK, Aland KV, Reid SA, Traub RJ, McCarthy JS, Lee R (2015). Emergence of neural angiostrongyliasis in eastern Australia. Vector Borne Zoonotic Dis.

[4] Morassutti AL, Thiengo SC, Fernandez M, Sawanyawisuth K, Graeff-Teixeira C (2014). Eosinophilic meningitis caused by *Angiostrongylus cantonensis*: an emergent disease in Brazil. Mem Inst Oswaldo Cruz.

[5] Traversa D, Di Cesare A, Conboy G (2010). Canine and feline cardiopulmonary parasitic nematodes in Europe: emerging and underestimated. Parasit Vectors.

[6] Helm JR, Morgan ER, Jackson MW, Wotton P, Bell R (2010). Canine angiostrongylosis: an emerging disease in Europe. J Vet Emerg Crit Care (San Antonio).

[7] Helm J, Roberts L, Jefferies R, Shaw SE, Morgan ER (2015). Epidemiological survey of *Angiostrongylus vasorum* in dogs and slugs around a new endemic focus in Scotland. Vet Rec.

[8] Taubert A, Pantchev N, Vrhovec MG, Bauer C, Hermosilla C (2009). Lungworm infections (*Angiostrongylus vasorum, Crenosoma vulpis, Aelurostrongylus abstrusus*) in dogs and cats in Germany and Denmark in 2003–2007. Vet Parasitol.

[9] Taylor CS, Garcia Gato R, Learmount J, Aziz NA, Montgomery C, Rose H (2015). Increased prevalence and geographic spread of the cardiopulmonary nematode *Angiostrongylus vasorum* in fox populations in Great Britain. Parasitology.

[10] Hermosilla C, Schug K, Hirzmann J, Schaper R, Taubert A. Lungworm (*Angiostrongylus vasorum, Crenosoma vulpis, Eucoleus aerophila*) infections in red fox populations in South West Germany (FEDAD 2014 Budapest). Abstracts. 2014;68.

[11] Giannelli A, Colella V, Abramo F, do Nascimento Ramos RA, Falsone L, Brianti E, et al. Release of lungworm larvae from snails in the environment: potential for alternative transmission pathways. PLoS Negl Trop Dis. 2015; doi:10.1371/journal.pntd.0003722. 10.1371/journal.pntd.0003722 PMC4401693 25884402

[12] Giannelli A, Cantacessi C, Colella V, Dantas-Torres F, Otranto D. Gastropod-borne helminths: A look at the snail-parasite interplay. Trends Parasitol 2015; doi:10.1016/j.pt.2015.12.002. 10.1016/j.pt.2015.12.002 26740470

[13] Rosen L, Ash LR, Wallace GD (1970). Life history of the canine lungworm *Angiostrongylus vasorum* (Baillet). Am J Vet Res.

[14] Yoshino TP, Wu XJ, Gonzalez LA, Hokke CH (2013). Circulating *Biomphalaria glabrata* hemocyte subpopulations possess shared schistosome glycans and receptors capable of binding larval glycoconjugates. Exp Parasitol.

[15] Cheng TC (1984). A classification of molluscan hemocytes based on functional evidences. Invertebrate Blood.

[16] Nakayama K, Nomoto AM, Nishijima M, Maruyama T (1997). Morphological and functional characterization of hemocytes in the giant clam *Tridacna crocea*. J Invertebr Pathol.

[17] Beck BH, Peatman E (2015). Mucosal ealth in aquaculture.

[18] Loker ES (2010). Gastropod immunobiology. Invertebrate Immunity.

[19] Sokolova IM (2009). Apoptosis in molluscan immune defense. Invertebrate Survival J.

[20] Matricon-Gondran M, Letocart M (1999). Internal defenses of the snail *Biomphalaria glabrata*: I. Characterization of hemocytes and fixed phagocytes. J Invertebr Pathol.

[21] Humphries JE, Yoshino TP (2003). Cellular receptors and signal transduction in molluscan hemocytes: connections with the innate immune system of vertebrates. Integr Comp Biol.

[22] Little TJ, Hultmark D, Read AF (2005). Invertebrate immunity and the limits of mechanistic immunology. Nat Immunol.

[23] Brinkmann V, Reichard U, Goosmann C, Fauler B, Uhlemann Y, Weiss DS (2004). Neutrophil extracellular traps kill bacteria. Science.

[24] Hermosilla C, Caro TM, Silva LM, Ruiz A, Taubert A (2014). The intriguing host innate immune response: novel anti-parasitic defence by neutrophil extracellular traps. Parasitology.

[25] Behrendt JH, Ruiz A, Zahner H, Taubert A, Hermosilla C (2010). Neutrophil extracellular trap formation as innate immune reactions against the apicomplexan parasite *Eimeria bovis*. Vet Immunol Immunopathol.

[26] Brinkmann V, Zychlinsky A (2012). Neutrophil extracellular traps: is immunity the second function of chromatin?. J Cell Biol.

[27] Branzk N, Lubojemska A, Hardison SE, Wang Q, Gutierrez MG, Brown GD, Papayannopoulos V (2014). Neutrophils sense microbe size and selectively release neutrophil extracellular traps in response to large pathogens. Nat Immunol.

[28] McCormick A, Heesemann L, Wagener J, Marcos V, Hartl D, Loeffler J (2010). NETs formed by human neutrophils inhibit growth of the pathogenic mold *Aspergillus fumigatus*. Microb Infect.

[29] Muñoz-Caro T, Rubio RM, Silva LM, Magdowski G, Gartner U, McNeilly TN (2015). Leucocyte-derived extracellular trap formation significantly contributes to *Haemonchus contortus* larval entrapment. Parasit Vectors.

[30] Nathan C (2006). Neutrophils and immunity: challenges and opportunities. Nat Rev Immunol.

[31] Urban CF, Ermert D, Schmid M, Abu-Abed U, Goosmann C, Nacken W (2009). Neutrophil extracellular traps contain calprotectin, a cytosolic protein complex involved in host defense against Candida albicans. PLoS Pathog.

[32] Parker H, Albrett AM, Kettle AJ, Winterbourn CC (2012). Myeloperoxidase associated with neutrophil extracellular traps is active and mediates bacterial killing in the presence of hydrogen peroxide. J Leukoc Biol.

[33] Reichel M, Muñoz-Caro T, Sanchez Contreras G, Rubio Garcia A, Magdowski G, Gartner U (2015). Harbour seal (*Phoca vitulina*) PMN and monocytes release extracellular traps to capture the apicomplexan parasite *Toxoplasma gondii*. Dev Comp Immunol.

[34] Hellenbrand KM, Forsythe KM, Rivera-Rivas JJ, Czuprynski CJ, Aulik NA (2013). *Histophilus somni* causes extracellular trap formation by bovine neutrophils and macrophages. Microb Pathog.

[35] Lin AM, Rubin CJ, Khandpur R, Wang JY, Riblett M, Yalavarthi S (2011). Mast cells and neutrophils release IL-17 through extracellular trap formation in psoriasis. J Immunol.

[36] Yousefi S, Gold JA, Andina N, Lee JJ, Kelly AM (2008). Catapult-like release of mitochondrial DNA by eosinophils contributes to antibacterial defense. Nat Med.

[37] Muñoz-Caro T, Silva LM, Ritter C, Taubert A, Hermosilla C (2014). *Besnoitia besnoiti* tachyzoites induce monocyte extracellular trap formation. Parasitol Res.

[38] Urban CF, Reichard U, Brinkmann V, Zychlinsky A (2006). Neutrophil extracellular traps capture and kill *Candida albicans* yeast and hyphal forms. Cell Microbiol.

[39] Jenne CN, Wong CH, Zemp FJ, McDonald B, Rahman MM, Forsyth PA (2013). Neutrophils recruited to sites of infection protect from virus challenge by releasing neutrophil extracellular traps. Cell Host Microbe.

[40] Silva LMR, Muñoz-Caro T, Burgos RA, Hidalgo MA, Taubert A, Hermosilla C. Far beyond phagocytosis: Phagocyte-derived extracellular traps act efficiently against protozoan parasites in vitro and in vivo. Mediators Inflamm. 2016; doi:10.1155/2016/5898074. 10.1155/2016/5898074 PMC4944069 27445437

[41] Chuah C, Jones MK, Burke ML, Owen HC, Anthony BJ, McManus DP (2013). Spatial and temporal transcriptomics of *Schistosoma japonicum*-induced hepatic granuloma formation reveals novel roles for neutrophils. J Leukoc Biol.

[42] Baker VS, Imade GE, Molta NB, Tawde P, Pam SD, Obadofin MO (2008). Cytokine-associated neutrophil extracellular traps and antinuclear antibodies in *Plasmodium falciparum* infected children under six years of age. Malar J.

[43] Silva LM, Muñoz-Caro TM, Gerstberger R, Vila-Vicosa MJ, Cortes HC, Hermosilla C, Taubert A (2014). The apicomplexan parasite *Eimeria arloingi* induces caprine neutrophil extracellular traps. Parasitol Res.

[44] Palic D, Ostojic J, Andreasen CB, Roth JA (2007). Fish cast NETs: neutrophil extracellular traps are released from fish neutrophils. Dev Comp Immunol.

[45] Chuammitri P, Ostojic J, Andreasen CB, Redmond SB, Lamont SJ, Palic D (2009). Chicken heterophil extracellular traps (HETs): novel defense mechanism of chicken heterophils. Vet Immunol Immunopathol.

[46] Poirier AC, Schmitt P, Rosa RD, Vanhove AS, Kieffer-Jaquinod S, Rubio TP (2014). Antimicrobial histones and DNA traps in invertebrate immunity: evidences in *Crassostrea gigas*. J Biol Chem.

[47] Robb CT, Dyrynda EA, Gray RD, Rossi AG, Smith VJ (2014). Invertebrate extracellular phagocyte traps show that chromatin is an ancient defence weapon. Nat Commun.

[48] Ng TH, Chang SH, Wu MH, Wang HC (2013). Shrimp hemocytes release extracellular traps that kill bacteria. Dev Comp Immunol.

[49] Ng TH, Wu MH, Chang SH, Aoki T, Wang HC (2015). The DNA fibers of shrimp hemocyte extracellular traps are essential for the clearance of *Escherichia coli*. Dev Comp Immunol.

[50] Graeff-Teixeira C, Geiger S, Walderich B, Hoffmann W, Abrahams E, Schulz-Key H (1999). Isolation of *Angiostrongylus costaricensis* first-stage larvae from rodent feces on a Percoll gradient. Parasitol Res.

[51] Barcante JM, Barcante TA, Dias SR, Vieira LQ, Lima WS, Negrao-Correa D (2003). A method to obtain axenic *Angiostrongylus vasorum* first-stage larvae from dog feces. Parasitol Res.

[52] Stoepler TM, Castillo JC, Lill JT, Eleftherianos I (2012). A simple protocol for extracting hemocytes from wild caterpillars. J Vis Exp.

[53] Patel Z, Gill AC, Fox MT, Hermosilla C, Backeljau T, Breugelmans K (2014). Molecular identification of novel intermediate host species of *Angiostrongylus vasorum* in Greater London. Parasitol Res.

[54] Cooper JE (1994). Bleeding of pulmonate snails. Lab Anim.

[55] Schauer C, Janko C, Munoz LE, Zhao Y, Kienhofer D, Frey B (2014). Aggregated neutrophil extracellular traps limit inflammation by degrading cytokines and chemokines. Nat Med.

[56] Hakkim A, Fuchs TA, Martinez NE, Hess S, Prinz H, Zychlinsky A, Waldmann H (2011). Activation of the Raf-MEK-ERK pathway is required for neutrophil extracellular trap formation. Nat Chem Biol.

[57] Martinelli S, Urosevic M, Daryadel A, Oberholzer PA, Baumann C, Fey MF (2004). Induction of genes mediating interferon-dependent extracellular trap formation during neutrophil differentiation. J Biol Chem.

[58] Lippolis JD, Reinhardt TA, Goff JP, Horst RL (2006). Neutrophil extracellular trap formation by bovine neutrophils is not inhibited by milk. Vet Immunol Immunopathol.

[59] Allam B: Rôle des fluides extrapalléaux des bivalves dans la défense immunitaire: cas de la maladie de l’anneau brun chez la palourde d’élevage, *Ruditapes philippinarum.* Doctoral dissertation. 1998

[60] Allam B, Paillard C (1998). Defense factors in clam extrapallial fluids. Dis Aquat Organ.

[61] Bachère E, Rosa RD, Schmitt P, Poirier AC, Merou N, Charrière GM, Destoumieux-Garzón D (2015). The new insights into the oyster antimicrobial defense: Cellular, molecular and genetic view. Fish Shellfish Immunol.

[62] Koiwai K, Alenton RRR, Kondo H, Hirono I. Extracellular trap formation in kuruma shrimp (Marsupenaeus japonicus) hemocytes is coupled with c-type lysozyme. Fish Shellfish Immunol. 2016;52:206–9. 10.1016/j.fsi.2016.03.039 27012393

[63] Abi Abdallah DS, Denkers EY (2012). Neutrophils cast extracellular traps in response to protozoan parasites. Front Immunol.

[64] Muñoz-Caro T, Hermosilla C, Silva LM, Cortes H, Taubert A (2014). Neutrophil extracellular traps as innate immune reaction against the emerging apicomplexan parasite *Besnoitia besnoiti*. PLoS One.

[65] Muñoz-Caro T, Mena Huertas SJ, Conejeros I, Alarcon P, Hidalgo MA, Burgos RA (2015). *Eimeria bovis*-triggered neutrophil extracellular trap formation is CD11b-, ERK 1/2-, p38 MAP kinase- and SOCE-dependent. Vet Res.

[66] Guimarães-Costa AB, DeSouza-Vieira TS, Paletta-Silva R, Freitas-Mesquita AL, Meyer-Fernandes JR, Saraiva EM (2014). 3′-nucleotidase/nuclease activity allows *Leishmania* parasites to escape killing by neutrophil extracellular traps. Infect Immun.

[67] Mendonça CL, Carvalho OS, Mota EM, Pelajo-Machado M, Caputo LF, Lenzi HL (1999). Penetration sites and migratory routes of *Angiostrongylus costaricensis* in the experimental intermediate host (*Sarasinula marginata*). Mem Inst Oswaldo Cruz.

[68] Muñoz-Caro T, da Silva LM R, Rentería-Solis Z, Taubert A, Hermosilla C (2016). Neutrophil extracellular traps in the intestinal mucosa of *Eimeria*-infected animals. Asian Pac J Trop Biomed.

[69] Guimaraes-Costa AB, Nascimento MT, Froment GS, Soares RP, Morgado FN, Conceicao-Silva F, Saraiva EM (2009). *Leishmania amazonensis* promastigotes induce and are killed by neutrophil extracellular traps. Proc Natl Acad Sci U S A.

[70] Munoz-Caro T, Lendner M, Daugschies A, Hermosilla C, Taubert A (2015). NADPH oxidase, MPO, NE, ERK1/2, p38 MAPK and Ca2+ influx are essential for *Cryptosporidium parvum*-induced NET formation. Dev Comp Immunol.

[71] Bonne-Annee S, Kerepesi LA, Hess JA, Wesolowski J, Paumet F, Lok JB (2014). Extracellular traps are associated with human and mouse neutrophil and macrophage mediated killing of larval *Strongyloides stercoralis*. Microbes Infect.

[72] Ferdushy T, Kapel CM, Webster P, Al-Sabi MN, Gronvold J (2009). The occurrence of *Angiostrongylus vasorum* in terrestrial slugs from forests and parks in the Copenhagen area, Denmark. J Helminthol.

[73] Wheeler ML, Underhill DM (2014). Time to cast a larger net. Nat Immunol.

[74] Tischendorf FW, Brattig NW, Büttner DW, Pieper A, Lintzel M (1996). Serum levels of eosinophil cationic protein, eosinophil-derived neurotoxin and myeloperoxidase in infections with filariea and schistosomes. Acta Trop.

