# Supplementary material for: Gastropod-derived haemocyte extracellular traps entrap metastrongyloid larval stages of Angiostrongylus vasorum, Aelurostrongylus abstrusus and Troglostrongylus brevior
Source: Parasit Vectors. 2017 Jan 31;10:50. doi: 10.1186/s13071-016-1961-z (PMC5282800; doi:10.1186/s13071-016-1961-z)
Supplement: Additional file 2: Figure S1. — Metastrongyloid larvae of Troglostrongylus brevior and Aelurostrongylus abstrusus attacked by mollusc haemocytes. Early ET formation against L1 of Troglostrongylus brevior confronted to haemocytes of Limax maximus analysed with contrast phase microscopy. (DOCX 376 kb) [file 13071_2016_1961_MOESM2_ESM.docx]

Further species of metastrongyloid larvae attacked by mollusc haemocytes:


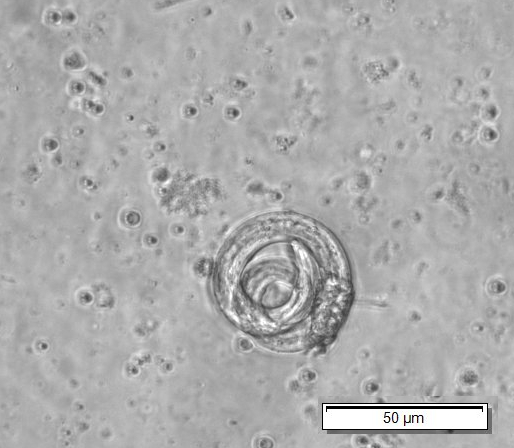


**Early ET formation against L1 of *Troglostrongylus brevior* confronted to haemocytes of *Limax maximus* analysed with contrast phase microscopy**

Arrows indicate haemocytes attacking the lungworm larvae after 30 min of incubation at RT.
