# Supplementary material for: Gastropod-derived haemocyte extracellular traps entrap metastrongyloid larval stages of Angiostrongylus vasorum, Aelurostrongylus abstrusus and Troglostrongylus brevior
Source: Parasit Vectors. 2017 Jan 31;10:50. doi: 10.1186/s13071-016-1961-z (PMC5282800; doi:10.1186/s13071-016-1961-z)
Supplement: Additional file 3: Figure S2. — Early ET formation against L1 of Aelurostrongylus abstrusus confronted to haemocytes of Limax maximus analysed with contrast phase microscopy. (DOCX 1533 kb) [file 13071_2016_1961_MOESM3_ESM.docx]

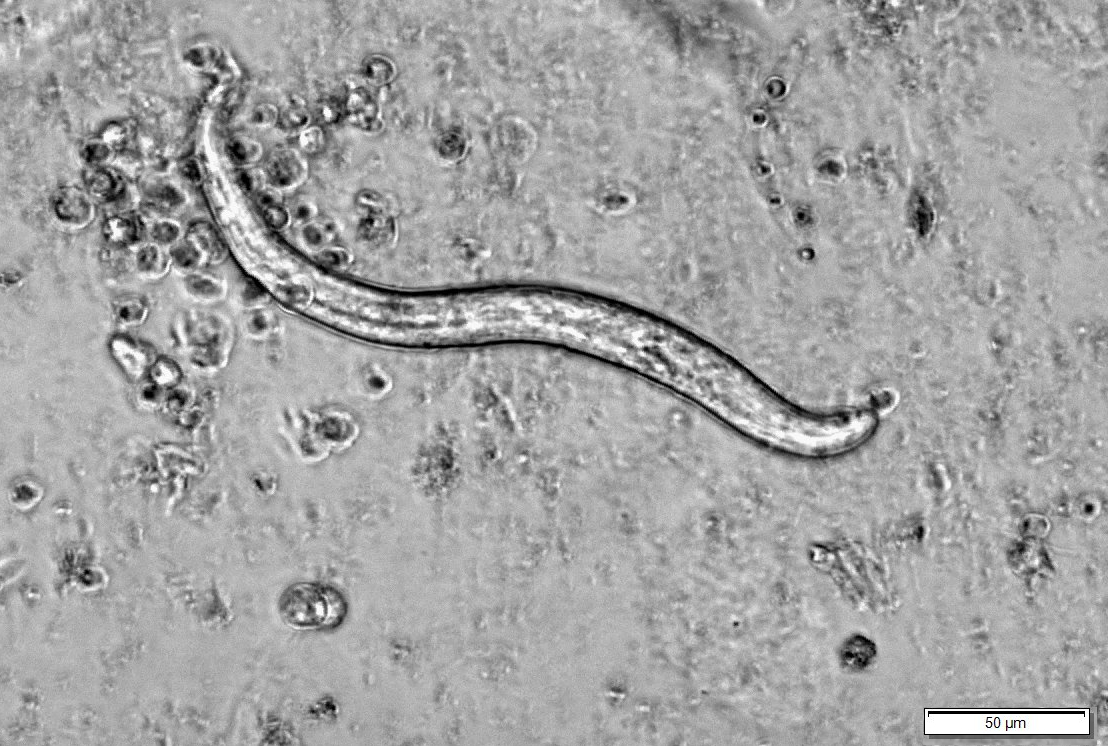


**Early ET formation against L1 of *Aelurostrongylus abstrusus* confronted to haemocytes of *Limax maximus* analysed with contrast phase microscopy**

Arrows indicate haemocytes attacking the lungworm larvae after 30 min of incubation at RT.
